# Supplementary material for: Comparison of In Vitro Methods for Assaying the Antibacterial Activity of a Mix of Natural Essential Oils Against Zoonotic Bacteria
Source: Microorganisms. 2025 May 14;13(5):1125. doi: 10.3390/microorganisms13051125 (PMC12114583; doi:10.3390/microorganisms13051125)
Supplement: Supplementary file 1 [file microorganisms-13-01125-s001.zip › microorganisms-3541982-supplementary.pdf]

Chromatogram and Results

Injection Details

Injection Name:	**Noyau huiles essentielles**	*Run Time (min):*	**56.94**
Vial Number:	**161**	*Injection Volume:*	**0.50**
Injection Type:	**Unknown**	*Channel:*	**TIC**
Calibration Level:		*Wavelength:*	**n.a.**
Instrument Method:	**GC FFAC full characterisation method split 200**	*Bandwidth:*	**n.a.**
Processing Method:		*Dilution Factor:*	**1.0000**
Injection Date/Time:	**26/Mar/25 12:54**	*Sample Weight:*	**1.0000**

Chromatogram

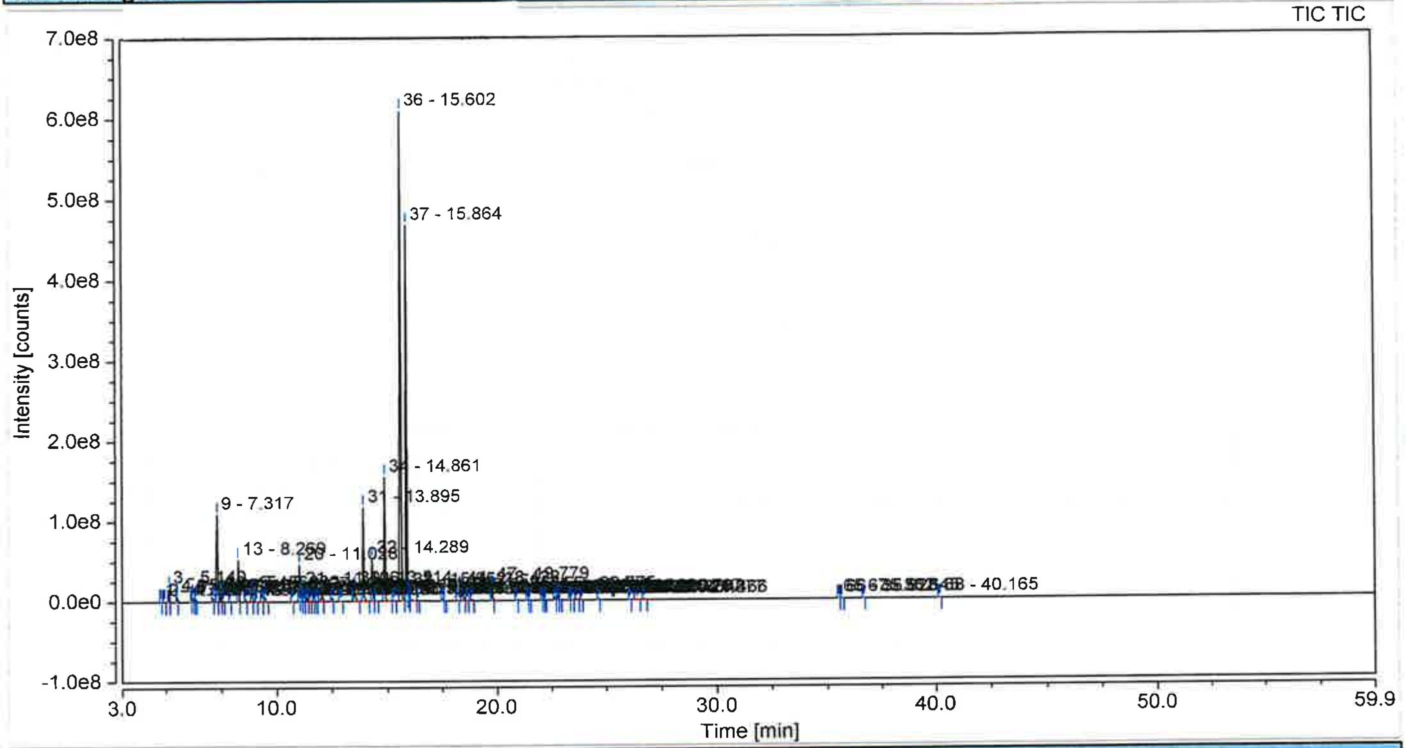

Integration Results

No.	Peak Name	Retention Time<br>min	Area<br>counts*min	Rel.Area<br>%
1		4.756	25301.438	0.03
2		4.926	43053.706	0.05
3	α pinene	5.140	471575.482	0.54
4	camphene	5.514	204743.567	0.23
5		6.157	110389.031	0.13
6		6.201	209337.192	0.24
7		6.354	131283.676	0.15
8		7.120	163034.160	0.19
9	p-cymène	7.317	3831377.726	4.39
10	Limonene	7.456	352681.142	0.40
11		7.579	43157.050	0.05
12		7.892	33200.131	0.04
13	γ-terpinene	8.269	1873751.200	2.15
14		8.599	99682.282	0.11
15	Diallyl disulfide	8.882	366128.917	0.42
16		9.082	16565.874	0.02
17		9.320	28132.315	0.03
18	Linalol	9.463	262944.570	0.30
19		10.698	66804.393	0.08
20	Isopulegol	11.028	1831727.686	2.10
21	Citronellal	11.106	613354.528	0.70

Chromeleon (c) Dionex
Version 7.2.9.20010

22		11.262	51081.182	0.06
23	Neo Isopulegol	11.358	270443.418	0.31
24		11.517	33511.607	0.04
25		11.704	58379.945	0.07
26		11.786	88969.356	0.10
27	Terpinen-4-ol	12.058	377272.802	0.43
28	α-terpineol	12.514	176935.350	0.20
29		12.905	29907.781	0.03
30	Citronellol	13.514	697287.195	0.80
31	β-citral	13.895	5083651.434	5.83
32	Geraniol	14.289	2294235.045	2.63
33		14.425	62455.401	0.07
34	α-citral	14.861	6983071.554	8.01
35		15.299	59486.303	0.07
36	Thymol	15.602	32896643.257	37.72
37	Carvacrol	15.864	23333235.239	26.76
38	Diallyl disulfide	15.952	601832.260	0.69
39		16.017	154641.825	0.18
40		16.381	25540.406	0.03
41		17.517	249111.719	0.29
42		17.568	1079.292	0.00
43		18.187	62627.096	0.07
44		18.422	628231.228	0.72
45		18.653	23503.610	0.03
46		18.830	90558.420	0.10
47	Caryophyllene	19.779	848372.534	0.97
48		20.870	31642.323	0.04
49		21.377	17743.815	0.02
50		21.466	32202.227	0.04
51		22.020	30465.534	0.03
52		22.105	32714.344	0.04
53		22.187	37786.791	0.04
54		22.615	183261.235	0.21
55		22.775	277257.291	0.32
56		22.857	18155.937	0.02
57		23.306	20243.571	0.02
58		23.404	37041.426	0.04
59		23.632	105047.258	0.12
60		23.857	23146.609	0.03
61		24.629	100528.271	0.12
62		26.047	16273.345	0.02
63		26.384	51397.674	0.06
64		26.707	108113.309	0.12
65		35.502	32711.430	0.04
66		35.628	25604.741	0.03
67		36.645	27786.091	0.03
68		40.165	35666.436	0.04
**Total:**			**87205080.981**	
