# Supplementary material for: Comparison of In Vitro Methods for Assaying the Antibacterial Activity of a Mix of Natural Essential Oils Against Zoonotic Bacteria
Source: Microorganisms. 2025 May 14;13(5):1125. doi: 10.3390/microorganisms13051125 (PMC12114583; doi:10.3390/microorganisms13051125)
Supplement: Supplementary file 1 [file microorganisms-13-01125-s001.zip › microorganisms-3541982-Table S1.pdf]

EO mix concentration (µL/mL)	*P. aeruginosa* lag phase ±SD (H)	*P. aeruginosa* µmax ±SD (H-1)	*P. aeruginosa* OD max ±SD
5,00E+03	0,42±0,09*	1,03±0,20*	0,99±0,11*
2,50E+03	0,39±0,11*	1,39±0,24*	1,24±0,08
1,25E+03	0,32±0,07	1,28±0,28*	1,24±0,31*
6,25E+02	0,29±0,10	1,35±0,40*	1,11±0,14*
3,13E+02	0,25±0,03	1,57±0,19*	1,18±0,11*
1,56E+02	0,22±0,04	1,82±0,13*	1,02±0,06*
7,81E+01	0,22±0,04	2,07±0,24*	1,09±0,09*
3,91E+01	0,20±0,03	2,57±0,36	1,04±0,04*
1,95E+01	0,20±0,03	3,41±0,72	1,12±0,12*
9,77E+00	0,15±0,04*	3,91±0,68	1,25±0,10
4,88E+00	0,16±0,04*	4,01±0,60	1,17±0,09*
Control	0,23±0,02	7,51±2,51	1,62±0,07

*Table S1: P. aeruginosa growth parameters depending on EO mix concentration (µL/mL) ; Lag phase (H) µmax (H-1) and OD max values are means of 3 replicates ; Mean differences between EO mix concentrations and control were tested by Kruskal-Wallis test ; * : within a row, mean with * are significantly different from control p<0,05*
